# 2,6-Diazido­toluene

**DOI:** 10.1107/S160053680706744X

**Published:** 2008-01-04

**Authors:** Thomas M. Klapötke, Burkhard Krumm, Matthias Scherr, Gunnar Spiess

**Affiliations:** a Department of Chemistry and Biochemistry, Ludwig-Maximilian University, Butenandtstrasse 5–13, D-81377 Munich, Germany

## Abstract

The structure of the title compound, C_7_H_6_N_6_, consists of almost planar mol­ecules with C—N distances of 1.429 (2) and 1.428 (2) Å. The H atoms of the methyl group are disordered over two sites with occupancy factors of 0.69 and 0.31. The azide groups show typical geometry for covalently bound azides.

## Related literature

The preparation of the title compound by a slightly different procedure was reported by Chapyshev & Tomioka (2003 ▶). For the comparable compound, 2-azido­benzyl ­bromide, see: Klapötke *et al.* (2003 ▶).
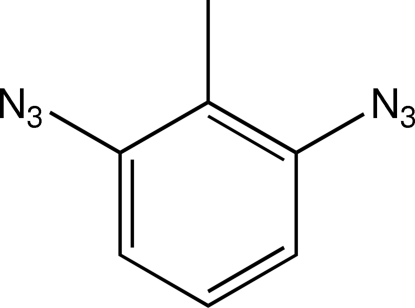


## Experimental

### 

#### Crystal data

C_7_H_6_N_6_
*M* *_r_* = 174.16
Orthorhombic, 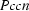

*a* = 12.298 (5) Å
*b* = 25.896 (5) Å
*c* = 5.085 (5) Å
*V* = 1619.4 (18) Å^3^
*Z* = 8
Mo *K*α radiation
μ = 0.10 mm^−1^
*T* = 200 (2) K
0.29 × 0.14 × 0.13 mm

#### Data collection

Oxford Xcalibur3 CCD area-detector diffractometer
Absorption correction: multi-scan (*CrysAlis RED*; Oxford Diffraction, 2007 ▶) *T*_min_ = 0.899, *T*_max_ = 0.990
5871 measured reflections
1578 independent reflections
956 reflections with *I* > 2σ(*I*)
*R*_int_ = 0.040

#### Refinement

*R*[*F* ^2^ > 2σ(*F* ^2^)] = 0.030
*wR*(*F* ^2^) = 0.061
*S* = 0.94
1578 reflections
143 parameters
H atoms treated by a mixture of independent and constrained refinement
Δρ_max_ = 0.10 e Å^−3^
Δρ_min_ = −0.12 e Å^−3^

### 

Data collection: *CrysAlis CCD* (Oxford Diffraction, 2007 ▶); cell refinement: *CrysAlis RED* (Oxford Diffraction, 2007 ▶); data reduction: *CrysAlis RED*; program(s) used to solve structure: *SIR97* (Altomare *et al*., 1999 ▶); program(s) used to refine structure: *SHELXL97* (Sheldrick, 1997 ▶); molecular graphics: *DIAMOND* (Brandenburg, 1996 ▶); software used to prepare material for publication: *SHELXL97*.

## Supplementary Material

Crystal structure: contains datablocks I, global. DOI: 10.1107/S160053680706744X/fj2092sup1.cif

Structure factors: contains datablocks I. DOI: 10.1107/S160053680706744X/fj2092Isup2.hkl

Additional supplementary materials:  crystallographic information; 3D view; checkCIF report

## Figures and Tables

**Fig. 1.**
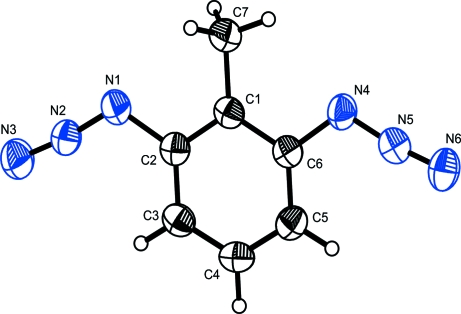
Molecular structure of C7H6N6 with displacement ellipsoids drawn at the 50% probability level. The minor disorder component of the methyl hydrogen atoms has been omitted.

## supplementary crystallographic information

### Comment

The structure of the title compound exhibits C–N distances [C2–N1 1.429 (2) and C6–N4 1.428 (2) Å] similar to the distance in 2-N_3_C_6_H_4_CH_2_Br [1.428 (5) Å, Klapötke *et al.*, 2003]. The values of both azide groups are in the common range for covalent azide groups with longer N_α_–N_β_ distances [N1–N2 1.241 (2) and N4–N5 1.253 (2) Å] and shorter terminal N_β_–N_γ_ distances [N2–N3 1.133 (2) and N5–N6 1.122 (2) Å] with more triple bond character. The azide angles are slightly bent [N1–N2–N3 172.98 (18) and N4–N5–N6 172.36 (17)°].

### Experimental

The title compound was prepared according the literature [Chapyshev & Tomioka (2003)], slightly modified, *e.g.* the column chromatography was performed with hexane/chloroform (1:4) as an eluent. Colorless crystals were obtained by slow evaporation of a chloroform solution. ^1^H NMR (400 MHz, CDCl_3_, Me_4_Si): δ 7.23 (tq, 4-H, ^3^J_H–H_ = 8.1 Hz, ^6^J_H—H_ = 0.5 Hz, 1H), 6.89 (d, 3-H, 2H), 2.05 (m, CH_3_, 3H) p.p.m.; ^13^C NMR (400 MHz, CDCl_3_, Me_4_Si): δ 140.0/127.2/121.3/114.0 (Ar—C), 11.2 (CH_3_) p.p.m.; ^15^N NMR (400 MHz, CDCl_3_, MeNO_2_) δ -139.5 (N_β_), -149.0 (N_γ_), -291.4 (N_α_) p.p.m..

### Crystal data

**Table:**

C_7_H_6_N_6_	*F*_000_ = 720
*M**_r_* = 174.16	*D*_x_ = 1.429 (1) Mg m^−^^3^
Orthorhombic, *P**c**c**n*	Mo *K*α radiation λ = 0.71073 Å
Hall symbol: -P 2ab 2ac	Cell parameters from 1382 reflections
*a* = 12.298 (5) Å	θ = 3.9–30.0º
*b* = 25.896 (5) Å	µ = 0.10 mm^−^^1^
*c* = 5.085 (5) Å	*T* = 200 (2) K
*V* = 1619.4 (18) Å^3^	Needle, colorless
*Z* = 8	0.29 × 0.14 × 0.13 mm

### Data collection

**Table:**

Oxford Xcalibur3 CCD area-detector diffractometer	1578 independent reflections
Radiation source: fine-focus sealed tube	956 reflections with *I* > 2σ(*I*)
Monochromator: graphite	*R*_int_ = 0.040
Detector resolution: 15.9809 pixels mm^-1^	θ_max_ = 26.0º
*T* = 200(2) K	θ_min_ = 4.3º
ω scans	*h* = −15→9
Absorption correction: multi-scan(CrysAlis RED; Oxford Diffraction, 2007)	*k* = −19→31
*T*_min_ = 0.899, *T*_max_ = 0.990	*l* = −5→6
5871 measured reflections	

### Refinement

**Table:**

Refinement on *F*^2^	Secondary atom site location: difference Fourier map
Least-squares matrix: full	Hydrogen site location: inferred from neighbouring sites
*R*[*F*^2^ > 2σ(*F*^2^)] = 0.030	H atoms treated by a mixture of independent and constrained refinement
*wR*(*F*^2^) = 0.061	*w* = 1/[σ^2^(*F*_o_^2^) + (0.0396*P*)^2^] where *P* = (*F*_o_^2^ + 2*F*_c_^2^)/3
*S* = 0.94	(Δ/σ)_max_ < 0.001
1578 reflections	Δρ_max_ = 0.11 e Å^−^^3^
143 parameters	Δρ_min_ = −0.11 e Å^−^^3^
Primary atom site location: structure-invariant direct methods	Extinction correction: none

### Special details

**Table:**

Refinement. Aromatic H atoms were placed in idealized positions and allowed to ride on their respective parent atoms, with C–H = 0.95 (C_arom_H) and with *U*_iso_(H) = k*U*_eq_(carrier atom), where k = 1.2 for CH. The H atoms attached to methyl C7 are disordered over two sites. These were freely refined; the occupancy of the major disorder component is 0.69 (5). The highest peak and deepest hole in the final difference map were located 0.93 Å from atom H7B and 0.32 Å from atom H7A, respectively.

### Fractional atomic coordinates and isotropic or equivalent isotropic displacement parameters (Å^2^)

**Table:**

	*x*	*y*	*z*	*U*_iso_*/*U*_eq_	Occ. (<1)
C1	0.45618 (11)	0.14719 (5)	0.0665 (3)	0.0348 (4)	
C2	0.45326 (12)	0.10499 (5)	−0.1031 (3)	0.0353 (4)	
C3	0.53072 (12)	0.06637 (6)	−0.0938 (3)	0.0415 (4)	
H3	0.5271	0.0383	−0.2136	0.050*	
C4	0.61302 (13)	0.06857 (6)	0.0888 (3)	0.0426 (4)	
H4	0.6662	0.0420	0.0957	0.051*	
C5	0.61819 (12)	0.10957 (5)	0.2622 (3)	0.0405 (4)	
H5	0.6745	0.1111	0.3899	0.049*	
C6	0.54110 (12)	0.14841 (5)	0.2495 (3)	0.0353 (4)	
C7	0.37338 (17)	0.18935 (8)	0.0524 (5)	0.0462 (4)	
H7A	0.297 (2)	0.1729 (9)	0.075 (5)	0.042 (10)*	0.69 (5)
H7B	0.385 (3)	0.2132 (11)	0.190 (8)	0.073 (11)*	0.69 (5)
H7C	0.381 (3)	0.2054 (10)	−0.129 (7)	0.060 (11)*	0.69 (5)
H7D	0.323 (4)	0.1895 (16)	−0.102 (10)	0.010 (16)*	0.31 (5)
H7E	0.334 (4)	0.193 (2)	0.231 (14)	0.04 (2)*	0.31 (5)
H7F	0.410 (4)	0.2239 (18)	0.031 (10)	0.015 (18)*	0.31 (5)
N1	0.36418 (10)	0.10504 (5)	−0.2837 (2)	0.0440 (4)	
N2	0.35797 (10)	0.06789 (5)	−0.4371 (3)	0.0419 (3)	
N3	0.34235 (11)	0.03628 (5)	−0.5865 (3)	0.0564 (4)	
N4	0.54156 (10)	0.19271 (4)	0.4164 (3)	0.0440 (3)	
N5	0.61378 (11)	0.19404 (4)	0.5897 (3)	0.0409 (3)	
N6	0.67279 (12)	0.19998 (5)	0.7553 (3)	0.0528 (4)	

### Atomic displacement parameters (Å^2^)

**Table:**

	*U*^11^	*U*^22^	*U*^33^	*U*^12^	*U*^13^	*U*^23^
C1	0.0329 (8)	0.0333 (8)	0.0382 (9)	−0.0034 (8)	0.0065 (8)	0.0026 (8)
C2	0.0346 (9)	0.0381 (8)	0.0332 (9)	−0.0052 (8)	0.0037 (8)	0.0030 (8)
C3	0.0440 (9)	0.0367 (8)	0.0436 (10)	0.0011 (9)	0.0053 (9)	−0.0017 (8)
C4	0.0415 (9)	0.0384 (8)	0.0480 (10)	0.0065 (9)	0.0043 (10)	0.0052 (9)
C5	0.0379 (9)	0.0444 (9)	0.0393 (9)	−0.0016 (9)	−0.0016 (9)	0.0050 (8)
C6	0.0367 (9)	0.0352 (8)	0.0340 (8)	−0.0039 (8)	0.0038 (8)	0.0013 (8)
C7	0.0428 (11)	0.0422 (10)	0.0537 (13)	0.0029 (11)	−0.0036 (12)	−0.0064 (12)
N1	0.0455 (8)	0.0406 (7)	0.0460 (8)	−0.0019 (7)	−0.0033 (7)	−0.0088 (7)
N2	0.0408 (8)	0.0462 (8)	0.0387 (8)	−0.0031 (8)	−0.0005 (7)	0.0000 (8)
N3	0.0611 (9)	0.0572 (9)	0.0510 (9)	−0.0003 (8)	−0.0051 (8)	−0.0148 (8)
N4	0.0455 (8)	0.0477 (8)	0.0388 (8)	−0.0006 (7)	−0.0065 (8)	−0.0063 (7)
N5	0.0455 (8)	0.0416 (8)	0.0357 (8)	−0.0052 (7)	0.0065 (8)	0.0007 (7)
N6	0.0545 (8)	0.0650 (10)	0.0389 (9)	−0.0080 (8)	−0.0047 (8)	−0.0028 (7)

### Geometric parameters (Å, °)

**Table:**

C1—C2	1.393 (2)	C6—N4	1.4270 (19)
C1—C6	1.399 (2)	C7—H7A	1.04 (3)
C1—C7	1.495 (2)	C7—H7B	0.94 (3)
C2—C3	1.382 (2)	C7—H7C	1.02 (3)
C2—N1	1.4294 (19)	C7—H7D	1.00 (5)
C3—C4	1.375 (2)	C7—H7E	1.04 (7)
C3—H3	0.9500	C7—H7F	1.01 (5)
C4—C5	1.382 (2)	N1—N2	1.2410 (17)
C4—H4	0.9500	N2—N3	1.1331 (17)
C5—C6	1.3836 (19)	N4—N5	1.2518 (19)
C5—H5	0.9500	N5—N6	1.1222 (17)
			
C2—C1—C6	116.67 (13)	H7A—C7—H7C	111 (2)
C2—C1—C7	121.72 (16)	H7B—C7—H7C	113 (2)
C6—C1—C7	121.60 (15)	C1—C7—H7D	118 (2)
C3—C2—C1	121.93 (15)	H7A—C7—H7D	62 (2)
C3—C2—N1	123.42 (13)	H7B—C7—H7D	132 (3)
C1—C2—N1	114.65 (14)	H7C—C7—H7D	49 (2)
C4—C3—C2	120.03 (15)	C1—C7—H7E	110 (2)
C4—C3—H3	120.0	H7A—C7—H7E	61 (3)
C2—C3—H3	120.0	H7B—C7—H7E	50 (3)
C3—C4—C5	119.80 (15)	H7C—C7—H7E	143 (3)
C3—C4—H4	120.1	H7D—C7—H7E	113 (3)
C5—C4—H4	120.1	C1—C7—H7F	110 (2)
C4—C5—C6	119.80 (14)	H7A—C7—H7F	141 (2)
C4—C5—H5	120.1	H7B—C7—H7F	55 (2)
C6—C5—H5	120.1	H7C—C7—H7F	60 (2)
C5—C6—C1	121.76 (13)	H7D—C7—H7F	101 (3)
C5—C6—N4	123.63 (14)	H7E—C7—H7F	103 (3)
C1—C6—N4	114.61 (13)	N2—N1—C2	116.77 (13)
C1—C7—H7A	108.3 (11)	N3—N2—N1	172.70 (16)
C1—C7—H7B	109.8 (17)	N5—N4—C6	116.34 (13)
H7A—C7—H7B	109 (3)	N6—N5—N4	172.29 (15)
C1—C7—H7C	106.0 (15)		
			
C6—C1—C2—C3	−0.6 (2)	C4—C5—C6—N4	−178.50 (14)
C7—C1—C2—C3	178.86 (16)	C2—C1—C6—C5	−0.2 (2)
C6—C1—C2—N1	179.09 (12)	C7—C1—C6—C5	−179.69 (16)
C7—C1—C2—N1	−1.5 (2)	C2—C1—C6—N4	179.16 (13)
C1—C2—C3—C4	0.8 (2)	C7—C1—C6—N4	−0.3 (2)
N1—C2—C3—C4	−178.84 (13)	C3—C2—N1—N2	0.2 (2)
C2—C3—C4—C5	−0.2 (2)	C1—C2—N1—N2	−179.43 (13)
C3—C4—C5—C6	−0.6 (2)	C5—C6—N4—N5	−3.5 (2)
C4—C5—C6—C1	0.8 (2)	C1—C6—N4—N5	177.14 (13)
